# Supplementary material for: Synthesis and photoluminescence properties of silica-modified SiO2@ANA-Si-Tb@SiO2, SiO2@ANA-Si-Tb-L@SiO2 core–shell–shell nanostructured composites
Source: R Soc Open Sci. 2019 Aug 7;6(8):190182. doi: 10.1098/rsos.190182 (PMC6731695; doi:10.1098/rsos.190182)
Supplement: Synthesis and Photoluminescence Properties of Silica-modified SiO2@ANA-Si-Tb@SiO2, SiO2@ANA-Si-Tb-L@SiO2 Core-Shell-Shell Nanostructured Composites [file rsos190182supp1.doc]

**[Supporting Information]**

**Synthesis and Photoluminescence Properties of Silica-modified** **SiO_2_@ANA-Si-Tb@SiO_2_, SiO_2_@ANA-Si-Tb-L@SiO_2_** **Core-Shell-Shell Nanostructured Composites**

Lina Feng ^1^, Wenxian Li ^1^,*, Jinrong Bao ^1^, Yushan Zheng ^2^, Yilian Li ^1^, Yangyang Ma ^1^, Kuisuo Yang ^1^, Yan Qiao ^1^, Anping Wu ^1^

^1^ Inner Mongolia Key Laboratory of Chemistry and Physics of Rare Earth Materials, School of Chemistry and Chemical Engineering, Inner Mongolia University, Hohhot 010021, PR China

^2^ Inner Mongolia Autonomous Region Food Inspection Test Center, Hohhot 010010, PR China


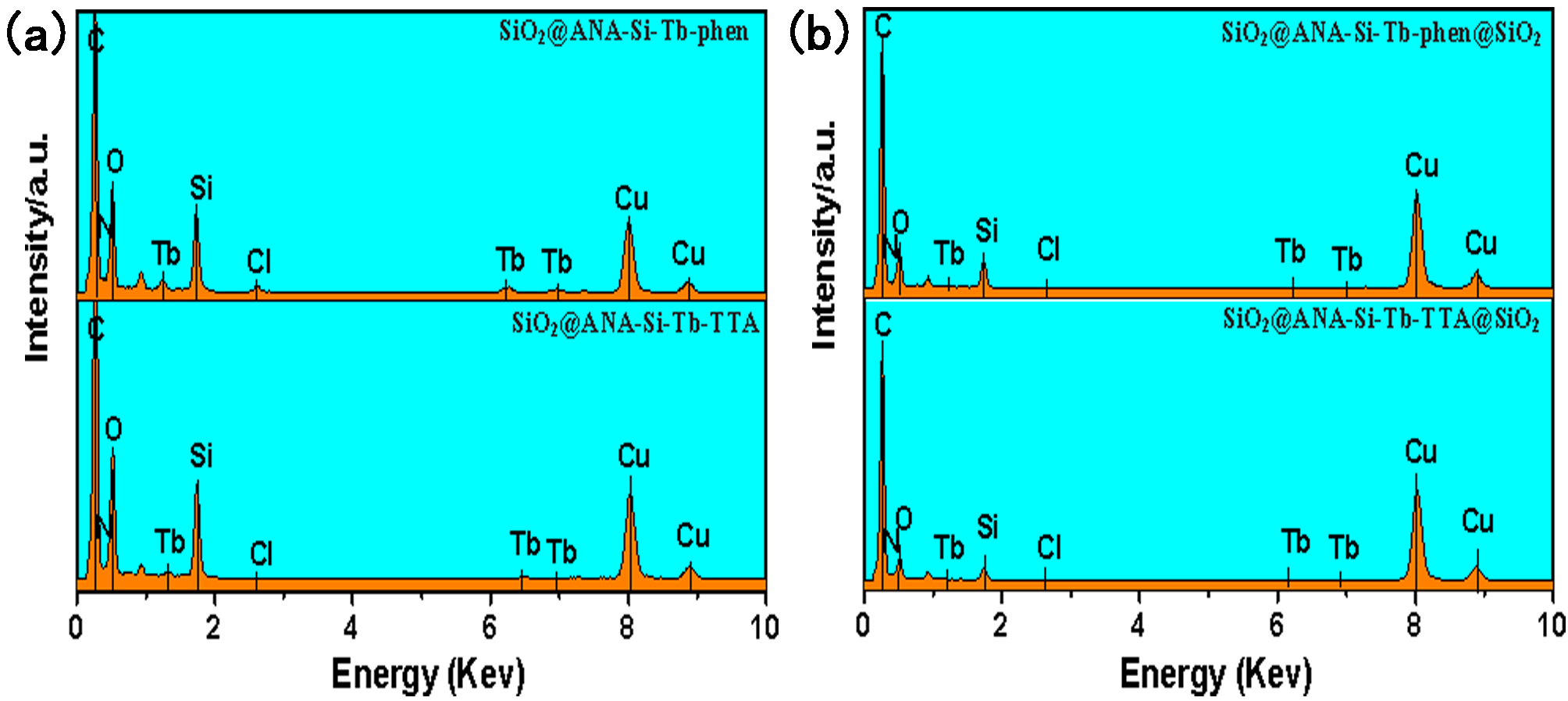


**Figure S1.** The EDX spectra of core-shell nanostructured composites (a) and core-shell-shell nanostructured composites (b).


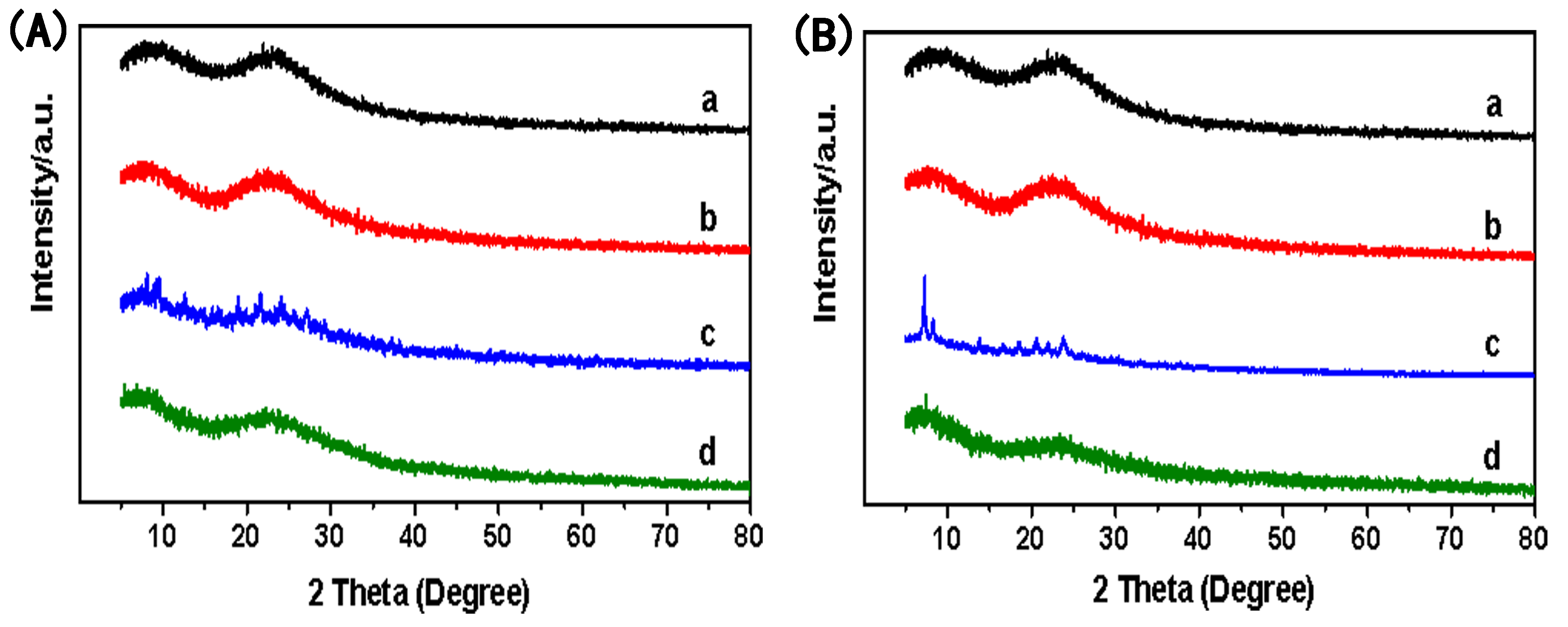


**Figure S2.** (A) XRD pattern of SiO_2_ (a), SiO_2_@ANA-Si (b), SiO_2_@ANA-Si-Tb-phen (c), SiO_2_@ANA-Si-Tb-phen@SiO_2_ (d). (B) XRD patterns of SiO_2_ (a), SiO_2_@ANA-Si (b), SiO_2_@ANA-Si-Tb-TTA (c), SiO_2_@ANA-Si-Tb-TTA@SiO_2_ (d).

**Table S1.** Photoluminescence emission spectra data of the Tb(III) core-shell and core-shell-shell nanostructured composites

| **Composites** | **Slit Width**  **(nm)** | **λ_EX_ (nm)** | **λ_EM_ (nm)** | **I (a.u.)** | **Energy**  **Transition** | **Intensity**  **Changes** |
| --- | --- | --- | --- | --- | --- | --- |
| SiO_2_@ANA-Si-Tb | 0.7 | 332 | 543 | 6，052，805 | ^5^D_4_→^7^F_5_ | - |
| SiO_2_@ANA-Si-Tb@SiO_2_ | 0.7 | 331 | 543 | 8，584，812 | ^5^D_4_→^7^F_5_ | 1.42 |
| SiO_2_@ANA-Si-Tb-phen | 0.7 | 322 | 543 | 12，700，600 | ^5^D_4_→^7^F_5_ | - |
| SiO_2_@ANA-Si-Tb-phen@SiO_2_ | 0.7 | 321 | 543 | 17，476，444 | ^5^D_4_→^7^F_5_ | 1.38 |
| SiO_2_@ANA-Si-Tb-TTA | 0.7 | 331 | 543 | 11，173，835 | ^5^D_4_→^7^F_5_ | - |
| SiO_2_@ANA-Si-Tb-TTA@SiO_2_ | 0.7 | 330 | 543 | 18，939，780 | ^5^D_4_→^7^F_5_ | 1.70 |


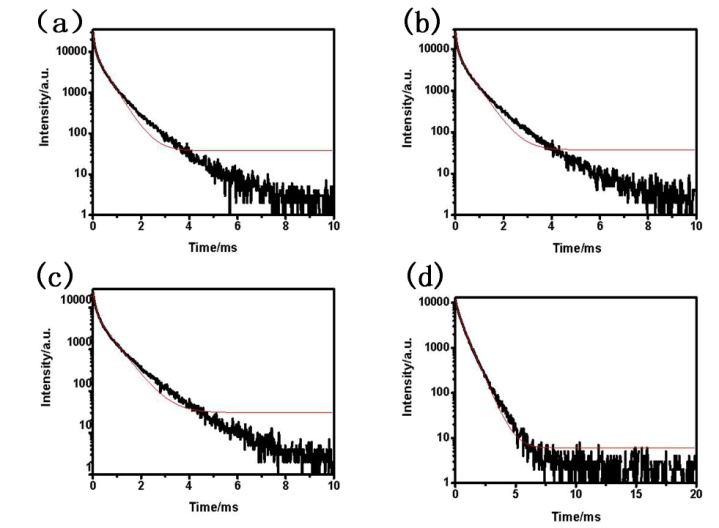


**Figure S3**. Lifetime curve of SiO_2_@ANA-Si-Tb (a), SiO_2_@ANA-Si-Tb@SiO_2_ (b), SiO_2_@ANA-Si-Tb-TTA (c) and SiO_2_@ANA-Si-Tb-TTA@SiO_2_ (d).

**Table S2.** The lifetimes parameters obtained from the biexponential fitting to the photoluminescence decay data of Tb(III) core-shell and core-shell-shell nanostructured composites

| **Composites** | **A_1_** | **A_2_** | **τ_1_ (ms)** | **τ_2_ (ms)** | **τ (µs)** | **R^2^** |
| --- | --- | --- | --- | --- | --- | --- |
| SiO_2_@ANA-Si-Tb | 19586.74 | 10377.72 | 0.0551 | 0.4481 | 374.03 | 0.99849 |
| SiO_2_@ANA-Si-Tb-phen | 3159.78 | 6091.30 | 0.2296 | 0.8392 | 763.44 | 0.99966 |
| SiO_2_@ANA-Si-Tb-TTA | 15784.78 | 7456.21 | 0.0863 | 0.5771 | 459.12 | 0.99904 |
| SiO_2_@ANA-Si-Tb@SiO_2_ | 29489.87 | 9187.42 | 0.0636 | 0.5020 | 375.24 | 0.99835 |
| SiO_2_@ANA-Si-Tb-phen@SiO_2_ | 2235.36 | 4917.75 | 0.2971 | 1.0627 | 976.40 | 0.99957 |
| SiO_2_@ANA-Si-Tb-TTA@SiO_2_ | 7393.76 | 4118.85 | 0.6932 | 0.1815 | 628.10 | 0.99967 |

**
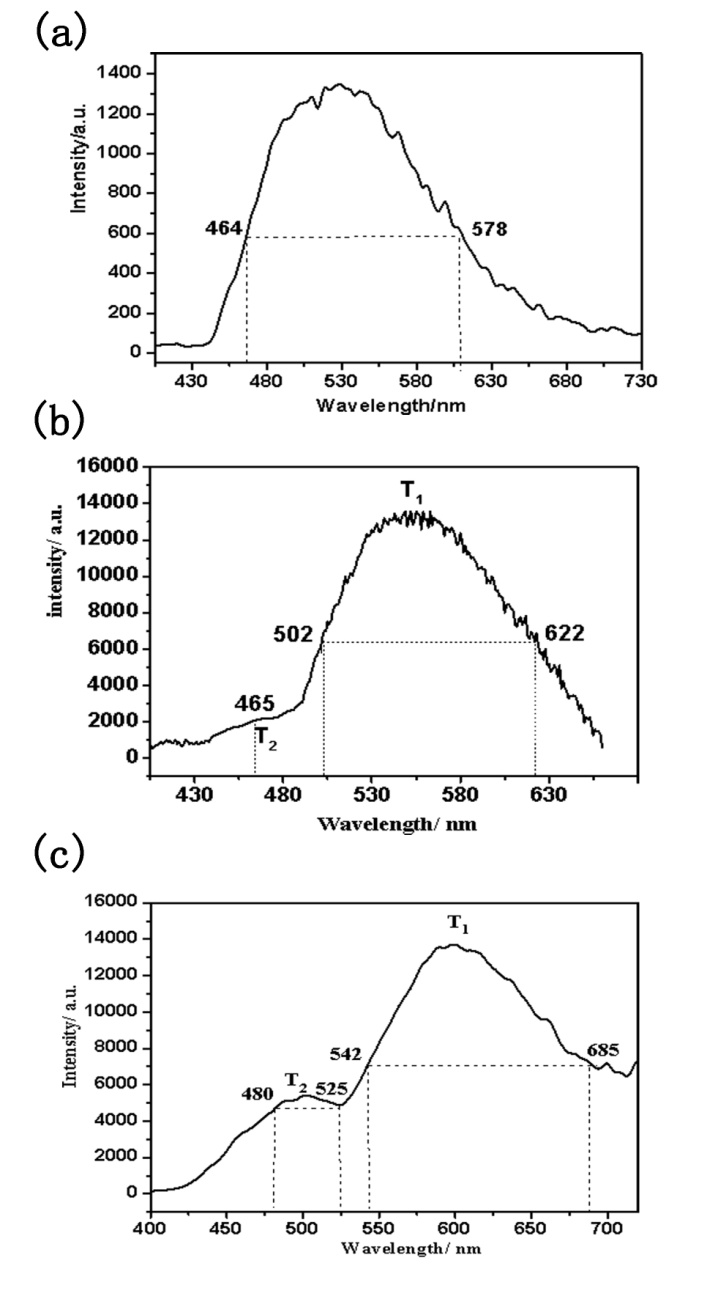
**

**Figure S4.** Low-temperature phosphorescence spectra of ANA-Si (a), phen (b) and TTA (c).
